# One-Year Update on Salivary Diagnostic of COVID-19

**DOI:** 10.3389/fpubh.2021.589564

**Published:** 2021-05-21

**Authors:** Douglas Carvalho Caixeta, Stephanie Wutke Oliveira, Leia Cardoso-Sousa, Thulio Marquez Cunha, Luiz Ricardo Goulart, Mario Machado Martins, Lina Maria Marin, Ana Carolina Gomes Jardim, Walter Luiz Siqueira, Robinson Sabino-Silva

**Affiliations:** ^1^Innovation Center in Salivary Diagnostic and Nanotheranostics, Department of Physiology, Institute of Biomedical Sciences, Federal University of Uberlandia, Uberlandia, Brazil; ^2^School of Dentistry, Federal University of Uberlandia, Uberlandia, Brazil; ^3^School of Medicine, Federal University of Uberlandia, Uberlandia, Brazil; ^4^Institute of Genetics and Biochemistry, Federal University of Uberlandia, Uberlandia, Brazil; ^5^College of Dentistry, University of Saskatchewan, Saskatoon, SK, Canada; ^6^Laboratory of Virology, Institute of Biomedical Science, Federal University of Uberlandia, Uberlandia, Brazil; ^7^São Paulo State University, Institute of Biosciences, Humanities and Exact Sciences, São José Do Rio Preto, Brazil

**Keywords:** nasopharyngeal swabs, saliva, diagnostic test, salivary diagnostic, SARS-CoV-2, COVID-19

## Abstract

**Background:** Coronavirus disease 2019 (COVID-19) is a global health problem, which is challenging healthcare worldwide. In this critical review, we discussed the advantages and limitations in the implementation of salivary diagnostic platforms of COVID-19. The diagnostic test of COVID-19 by invasive nasopharyngeal collection is uncomfortable for patients and requires specialized training of healthcare professionals in order to obtain an appropriate collection of samples. Additionally, these professionals are in close contact with infected patients or suspected cases of COVID-19, leading to an increased contamination risk for frontline healthcare workers. Although there is a colossal demand for novel diagnostic platforms with non-invasive and self-collection samples of COVID-19, the implementation of the salivary platforms has not been implemented for extensive scale testing. Up to date, several cross-section and clinical trial studies published in the last 12 months support the potential of detecting SARS-CoV-2 RNA in saliva as a biomarker for COVID-19, providing a self-collection, non-invasive, safe, and comfortable procedure. Therefore, the salivary diagnosis is suitable to protect healthcare professionals and other frontline workers and may encourage patients to get tested due to its advantages over the current invasive methods. The detection of SARS-CoV-2 in saliva was substantial also in patients with a negative nasopharyngeal swab, indicating the presence of false negative results. Furthermore, we expect that salivary diagnostic devices for COVID-19 will continue to be used with austerity without excluding traditional gold standard specimens to detect SARS-CoV-2.

## Introduction

The World Health Organization (WHO) indicates the pivotal importance of mass testing in the find–test–trace–isolate–support strategy to contain COVID-19 transmission (1). The increase in massive testing capacity coupled with artificial intelligence multidisciplinary data should be used to prevent and combat the negative effects of COVID-19 and strengthen global health public systems to improve COVID-19 response (2). The National Institute of Health supports a rapid scaling up of SARS-CoV-2-detecting tests in the United States (3); however, the need for better diagnostic tests with high sensitivity has been considered critical to mitigate and suppress the spread of COVID-19 (4). Novel continued efforts need to be performed to reduce the presence of false-negative molecular testing in presymptomatic and asymptomatic subjects, as well as the presence of hospitalized patients with initial false-negative testing and clinical signs and symptoms consistent with SARS-CoV-2 infection (4).

In this critical review, at the salivary SARS-CoV-2 detection's 1-year mark, we discussed the advantages and limitations in the implementation of salivary diagnosis of COVID-19 and point out some recommendations to this potential application to provide a comprehensive summary on the scientific advances performed in the last 12 months. The diagnostic test for COVID-19 by invasive nasopharyngeal collection is uncomfortable for the patients and requires specialized training of the frontline workers in order to perform an appropriate collection of samples. Additionally, these frontline professionals are in close contact with infected patients or suspected cases of COVID-19, leading to increased morbimortality of healthcare workers. It imposes the development of new strategies for COVID-19 diagnosis; however, despite the colossal demand for novel diagnostic platforms with non-invasive and self-collection samples of COVID-19, the accuracy of salivary SARS-CoV-2 platforms are still not well-elucidated. The pivotal impact on social, health, economic, and educational fields in a global emergency due to COVID-19 makes it more challenging to compare the advantages and limitations in implementing novel potential salivary platforms (1, 2, 4).

## Background

The coronavirus disease 2019 (COVID-19) is an international public health emergency, which also impacts social, economic, and educational aspects worldwide. The outbreak of COVID-19 is caused by the severe acute respiratory syndrome coronavirus 2 (SARS-CoV-2), which has been spread across all continents with more than 117 million cases and ~2.5 million deaths (5). The centers for disease control and prevention around the world have recommended testing for SARS-CoV-2 in upper respiratory specimens.

The COVID-19 diagnostic is mainly based on the detection of SARS-CoV-2 by real-time polymerase chain reaction (RT-PCR). The sensitivity of this gold standard test is higher in symptomatic than in asymptomatic COVID-19 subjects (6). Besides, the false-negative results have uncertain frequency especially in the incubation period of the disease. Although SARS-CoV-2 RNA detection in the nasopharyngeal swab was reported as the gold standard method for COVID-19 diagnosis, sample collection by this method requires that healthcare frontline workers are in close contact with infected patients or suspected cases of COVID-19. Besides, this specimen-collecting procedure is invasive and inconvenient for patients, and it requires specialized training for healthcare workers (7).

Salivary biomarkers are an alternative method to other invasive procedures in the early diagnostic of systemic diseases (8). The collection of saliva samples represents a non-invasive, convenient, and easy self-collection method, with no direct contact between healthcare workers and patients. Saliva contains more than 3,000 proteins, 3,000 mRNA, ~50 microRNAs, hundreds of metabolites, and more than 700 species of microorganisms such as viruses (9). The 198 extracellular RNAs (ExRNAs) detected in saliva were also considered potential biomarkers for systemic and oral diseases. In this context, salivary exRNA related to SARS-CoV-2 infection could be used to develop novel salivary platforms of COVID-19 (10, 11). Previously, we detailed the potential of salivary diagnosis for COVID-19 (12), which was confirmed in several studies by detecting SARS-CoV-2 in human saliva (13–37) and in saliva associated with oropharyngeal fluid (38, 39). SARS-CoV-2 was also detected in animal models of COVID-19. The viral RNA was detected in saliva and nasal washes from 2 to 8 days post-infection of infected ferrets as an animal model of COVID-19 (40). In this context, analysis of SARS-CoV-2 RNA from saliva can provide clues in the early diagnosis of COVID-19. Higher viral loads of SARS-CoV-2 in oropharyngeal fluid mixed with saliva were detected on symptom onset, which then gradually declined toward the detection limit until 25 days after symptoms started (39, 41).

## Future Directions

As the landscape of SARS-CoV-2 diagnosis comprises limitations for the current gold standard diagnosis methods and potential benefits for novel applications in COVID-19 diagnosis, a critical evaluation on the advantages and limitations of concurrent emerging salivary diagnosis is mandatory.

### Search Strategy and Study Selection

We searched three electronic databases, PubMed, LILACS, and Google Scholar, from February 2020 when the SARS-CoV-2 was first indicated in saliva (12, 38) until February 2021. The selected keywords were COVID-19, coronavirus, SARS-CoV-2, saliva, and nasopharyngeal swabs. We selected studies that analyzed the accuracy and sensitivity of saliva compared with nasopharyngeal swabs for SARS-CoV-2 detection by RT-PCR. Positive detection of SARS-CoV-2 in saliva or nasopharyngeal fluid was considered as a reference standard.

Table 1 compares the sensitivity of nasopharyngeal and salivary samples in SARS-CoV-2 RNA detection using RT-PCR and points out the presence of samples negative in nasopharyngeal swab and positive in saliva, which indicates the presence of false-negative results.

**Table 1:** Sensitivity of COVID-19 salivary diagnosis comparing to nasopharyngeal swab (NPS) specimens.

**References**	**Total samples**	**Positive (NPS+Saliva)**	**Negative**	**Sensitivity Saliva**	**Sensitivity NPS**	**Percentage of Saliva + and NPS -**
Rao et al. (30)	217	160	57	93.1%	52.5%	47.5%
				(149/160)	(84/160)	(76/160)
Caulley et al. (17)	272	13	259	84.6%	61.5%	38.5%
				(11/13)	(8/13)	(5/13)
Senok et al. (32)	401	35	366	80%	74.3%	25.7%
				(28/35)	(26/28)	(9/35)
Uwamino et al. (35)	196	58	138	74.1%	81%	19%
				(43/58)	(47/58)	(11/58)
Moreno-Contreras et al. (27)	71	34	37	73.5%	82.3	17.6%
				(25/34)	(28/34)	(6/34)
Yokota et al. (37)	1,924	52	1,872	92.3%	88.5%	11.5%
				(48/52)	(46/52)	(6/52)
Jamal et al. (21)	91	72	19	72.2%	88.9%	11.1%
				(52/72)	(64/72)	(8/72)
Iwasaki et al. (20)	76	10	66	90%	90%	10%
				(9/10)	(9/10)	(1/10)
Pasomsub et al. (28)	200	21	179	85.7%	90.5%	9.5%
				(18/21)	(19/21)	(2/21)
Torres et al. (34)	943	108	835	42.6%	92.6%	7.4%
				(46/108)	(100/108)	(8/108)
Hanson et al. (19)	354	86	268	94.2%	93%	7%
				(81/86)	(80/86)	(6/86)
Kandel et al. (22)	429	46	383	91.3%	93.5%	6.5%
				(42/46)	(43/46)	(3/46)
Landry et al. (23)	124	35	89	85.7%	94.3%	5.7%
				(30/35)	(33/35)	(2/35)
Babady et al. (14)	87	18	69	94.4%	94.4%	5.5%
				(17/18)	(17/18)	(1/18)
Miller et al. (26)^*^	91	36	55	97.2%	94.4%	5.5%
				(35/36)	(34/36)	(2/36)
Skolimowska et al. (33)	131	19	112	84.2%	94.7%	5.3%
				(16/19)	(18/19)	(1/19)
Altawalah et al. (13)	848	361	487	84.2%	95.3%	4.7%
				(304/361)	(344/361)	(17/361)
Matic et al. (24)	74	22	52	72.7%	95.4%	4.5%
				(16/22)	(21/22)	(1/22)
Chau et al. (18)	27	28	NA	75%	96.4%	3.6%
				(21/28)	(27/28)	(1/28)
Vaz et al. (36)	155	73	82	94.5%	97.3%	2.7%
				(69/73)	(71/73)	(2/73)
Barat et al. (15)	459	38	421	81.6%	97.4%	2.6%
				(31/38)	(37/38)	(1/38)
McCormick-Baw et al. (25)	156	50	106	96%	98%	2%
				(48/50)	(49/50)	(1/50)
Bhattacharya et al. (16)	74	58	16	91.4%	100%	0%
				(53/58)	(58/58)	(0/58)
Ranoa et al. (29)	100	9	91	100%	100%	0%
				(9/9)	(9/9)	(0/9)
Rutgers (31)	53	26	27	100%	100%	0%
				(26/26)	(26/26)	(0/26)
Overall	7,553	1,468	6,086	83.6%	88.4%	11.6%
				(1,227/1,468)	(1,298/1,468)	(171/1,468)

* *The specificity of both salivary and nasopharyngeal (NPS) swabs were 100%. Considering the presence of false-negative results with NPS, a positive detection of SARS-CoV-2 in saliva or nasopharyngeal fluid was considered as a positive standard reference*.

### Limitations of SARS-CoV-2 Detection in Nasopharyngeal Specimens

Although the nasopharyngeal swab tests have been considered as gold standard specimens, multisite assessment or other specimens for SARS-CoV-2 diagnosis have been suggested to reduce false-negative test results and to increase testing capacity (42), especially due to limitations in low–middle income countries (LMICs) (43). Nasopharyngeal collection is performed using a flexible plastic swab with a nylon tip, which is inserted into the nostrils until the healthcare worker observes resistance. Subsequently, the swab is rotated three times in the nasopharynx and removed after 5 s, a procedure which is considered invasive and uncomfortable (44, 45). However, the swab collection protocol can be different in each country. Appropriate nasopharyngeal swab collection is more difficult in children and patients with a deviated nasal septum or coagulopathy (46). The sputum is another respiratory specimen tested to be used in COVID-19 diagnosis. Due to the limitations of sampling, sputum collection was used in possibly only one third of COVID-19 patients, which reveals a robust restriction of this diagnostic method (12, 38). Self-collection of samples from suspected cases of COVID-19 or infected patients is still limited and the direct contact between healthcare workers and patients during the standard collection procedures resulted in about 20% of the healthcare workforce becoming infected, and some deaths were reported (47). Frontline workers may experience intense anxiety and additional adverse emotion due to the risk of contamination during the collection procedure (48). Although most studies showed higher levels of the virus in nasopharyngeal specimens compared with saliva, lower levels of SARS-CoV-2 in nasopharyngeal swabs can result in false-negative outcomes due to inaccurate collection (48). The personal protective equipment and creation of exclusive sampling rooms have been reported as tools capable of enhancing the protection of frontline workers (47, 48). Currently, COVID-19 cases have been significantly increasing worldwide, overloading national health systems. Furthermore, the situation might be even worse in LMICs, since there is a scarcity of trained healthcare and other frontline workers to face the COVID-19 pandemic (49). Taken together, these issues demonstrate the critical demand for new approaches for COVID-19 diagnosis.

### The Potential of Salivary Diagnosis of COVID-19

The enthusiasm in developing new salivary platforms for COVID-19 diagnosis and monitoring is comprehensible; however, the true accuracy of these new protocols to detect SARS-CoV-2 in saliva has been discussed in several scenarios such as during the incubation period, the viral response phase, and the host inflammatory phase of symptomatic patients. Besides, the diagnostic sensitivity levels in COVID-19 asymptomatic patients also remain unclear in both salivary and nasopharyngeal specimens. It is important to emphasize that the implementation of salivary diagnosis for COVID-19 before a comprehensive knowledge of its limitation could promote future issues about the application of salivary diagnostic tests to other systemic diseases. However, the colossal demand for novel diagnostic platforms for COVID-19 with non-invasive and self-collection samples could be used after the creation of a well-designed strategic plan for its implementation until this true efficacy will be completely investigated.

### Preponderance of Reviews and Letters Over Primary Clinical Trials

The most remarkable data on COVID-19 salivary diagnosis implementation is the unbalanced number of published clinical trials or reviews and letters. PubMed reveals 20 cross-sectional and case-control designed studies, five cross-sectional studies with no control subjects, and more than 200 reviews and letters published from February 2020 up to February 2021. Additionally, there are 14 additional cross-sectional studies that evaluated the oropharyngeal fluid mixed with saliva as a diagnostic fluid for COVID-19. It suggests that opinions concerning salivary diagnostic platforms have been consolidated primarily from letters and reviews. On the other hand, it is important to emphasize that cross-sectional studies with salivary diagnostics indicated a higher correlation of sensitivity compared with the gold standard nasopharyngeal samples in COVID-19 diagnosis. We performed this critical review due to the limitations concerning current reviews focusing on the counterbalance between the inevitable obstacles and encouraging results of COVID-19 salivary diagnosis.

### Sample Size of Studies

In order to obtain a more robust comparison of saliva with gold standard specimens, a limited number of comparative studies and lower sample sizes have been overcome in these 12 months after SARS-CoV-2 detection in saliva (38) Altogether, the total number of salivary samples (non-infected subjects and COVID-19 patients), which was compared with gold standard nasopharyngeal respiratory specimens was 7,553. In this context, 5,172 subjects were from published studies/original articles, 53 subjects from the FDA emergence-approved study, 191 subjects from preprint articles, 559 subjects from short communications/brief reports, and 1,578 subjects from letters to the editor. To detect the salivary sensitivity 1,468 salivary and/or NPS-positive samples from COVID-19-infected patients were used and evaluated in this review (Table 1).

### The Relevance of Specificity in SARS-CoV-2 Detection

In general, the absence of analysis in control subjects can be considered a negative condition; however, the main limitation in the use of RT-PCR tests is the detection of RNA in levels near the sensitivity limits. The detection of unspecific RNA is not a classical limitation of RT-PCR tests (50), which is considered 100% specific due to the intrinsic characteristics of this platform (51). It must be considered that the presence of SARS-CoV-2 RNA in saliva and negative results in nasopharyngeal samples analyzed by RT-PCR cannot be classified as false positive, but a misclassification of nasopharyngeal samples. This pivotal view is well-documented in a previous study that showed 71% of matched detection of SARS-CoV-2 RNA in saliva and nasopharyngeal swabs, 21% only in saliva, and 8% only in nasopharyngeal swabs (52). It can be related with the limitations in nasopharyngeal swab procedure and/or with low produced nasopharyngeal mucous secretion in COVID-19 patients. In this new pandemic era, the centers for disease control and prevention worldwide took maximal efforts to establish reference standards for COVID-19 diagnosis in a fast and efficient way, based on the outbreak of severe acute respiratory syndrome (SARS) in 2003 (53). It is well-recognized that updates in COVID-19 diagnosis protocols are crucial, and the reference standards are not perfect, especially in samples collected in the 1st days after infection (54). The procedures related to sample preservation and RNA extraction were reported in all included studies, and it seems suitable, and presumably these factors did not influence the results. In this context, it is important to emphasize that the absence of a control group in the studies with oropharyngeal fluid mixed with saliva is not a significant limitation (38, 39, 55–57). The reference standard using nasopharyngeal specimens was considered as an unsolved issue that needs imperative debate to increase confidence in COVID-19 tests (54).

### Saliva Collection and Its Correlation With Sensitivity

The pioneer study that detected viable SARS-CoV-2 in oral fluid promoted a paradigm shift in diagnosis, monitoring, and infection control for COVID-19 (38). However, the sensitivity of salivary SARS-CoV-2 RNA to diagnose COVID-19 needs to be carefully checked because some data are based on trials designed to evaluate oropharyngeal fluid mixed with saliva (38, 39, 57–59). In classical studies with salivary collection, the patient is not required to cough out fluid from their throat. Frequently, total saliva is collected from the mouth under an unstimulated or stimulated flow rate (9). Some collection devices were also developed to collect saliva specifically from parotid, submandibular/sublingual, and minor and palatine glands (9). Here, this review considered studies that collected saliva by the traditional drooling technique and without coughing into a container. Table 1 shows a similar sensitivity to detect SARS-CoV-2 RNA undergoing paired collection of saliva and NPS. SARS-CoV-2 RNA was detected overall in 83.6% (1,227/1,468) of saliva samples and also in 88.4% (1,298/1,468) of samples of NPS, which supports the potential of salivary SARS-CoV-2 RNA as a biomarker for COVID-19 in a preliminary analysis. We also highlight a substantial salivary detection of SARS-CoV-2 in patients with a negative nasopharyngeal swab (11.6%, 171/1,468), indicating an expressive indication of false-negative results with gold standard specimens. Thus, based on these data, we suggest that saliva is an accurate sample to be used for a mass screening test, and this biofluid could be used to reduce the rate of false negatives in the clinical performance of COVID-19 diagnostic tests. We also observed that the majority of articles analyzed unstimulated saliva, which avoids a potential dilution of the SARS-CoV-2, which could occur in mouth rinsing or stimulated saliva collection (60).

### The Importance of Home- and Self-Sample Collection

It was indicated that the primary choice for sampling during illness experience is home-based tests compared with clinic-based strategies. The higher compliance to test for SARS-CoV-2 was verified when a lower degree of contact with frontline healthcare workers was required to collect samples: as expected, home testing was the most preferred, followed by tests in drive-through sets and, subsequently, hospital-based testing. It is crucial to provide self-saliva collection and home-based tests to suspected cases of COVID-19 as profitable strategies in order to guarantee the social distance of the population. It also contributes to reduce direct contact with frontline workers, which offers a potential for early diagnosis due to the hierarchy of willingness to test for COVID-19. The self-sample collection and home-based tests should be validated as soon as possible to be applied in public and private healthcare systems (61).

### Spectrum of Patients

In order to provide a suitable spectrum of COVID-19 patients with distinct severity of diseases, it is important to envisage patients searching for a diagnostic test in the onset of symptoms and in the late stage of the disease. Bearing in mind that the higher salivary SARS-CoV-2 levels occur during the acute phase of disease with gradual decline after symptom onset (39), it is important to point out the limitations of longitudinal analysis with SARS-CoV-2 level in asymptomatic COVID-19 subjects. In this context, the temporal analysis of SARS-CoV-2 viral load in saliva should receive more attention among asymptomatic and non-hospitalized COVID-19 patients, which could be pivotal for the translation of salivary tests in the clinic. However, the current gold standard protocols are also unable to raise this query (54). The comparison between sensitivity shown in Table 1, in different studies, reported a limited heterogeneity, which should not be ignored to improve this new potential gold standard protocol.

### Obstacles to SARS-CoV-2 RNA Extraction From Saliva and New Applications

A critical hurdle for salivary diagnosis may be the broad-spectrum validation in COVID-19 patients during the incubation period, the viral response phase, and the host inflammatory phase in asymptomatic and symptomatic patients. It has been proposed that patients can be infected and after 24 to 72 hours the onset of symptoms could occur. About 50% of the transmission of cases is from asymptomatic COVID-19 individuals. The viral levels of SARS-CoV-2 RNA are presumably detected in nasopharyngeal swabs before or sooner than symptom onset, which is a leading challenge in the diagnosis, spread, and containment of COVID-19 (6).

Some critical issues in the isolation of RNA methods to process saliva are unique to this biofluid. The know-how and practice to pipette a biofluid with higher density could explain the discrepancy between the overall sensitivity in different studies. Some protocols indicated the dilution of saliva in a standard liquid as that occurring in nasopharyngeal swabs. This action can change the SARS-CoV-2 concentration and reduce the sensitivity of tests. In this context, the higher saliva density that makes pipetting difficult, tooth-brushing contaminants, and changes in volume are parameters that could interfere with the result (62). In general, the use of the magnetic bead methodology showed good results for saliva sensitivity, 97.2% (26), possibly due to the RNA extraction insulation kit used. In addition, the enzymes present in saliva also makes RNA naturally degrade, so choosing a more robust methodology is important for the sensitivity and specificity of the experiment (26).

Various methods are available to extract RNA from saliva, such as methods using phenol and guanidinium isothiocyanate, or commercially available silica membrane spin columns or magnetic bead-based RNA isolation kits (63). Other molecular diagnostic methods, such as reverse transcription–loop-mediated isothermal amplification (RT-LAMP), have also been reported as useful for diagnosing COVID-19 in settings of point-of care testing (64–66). Rapid and extraction-free detection of SARS-CoV-2 from saliva by colorimetric RT-LAMP is a simple, sensitive, rapid, and cost-effective approach with broad potential to expand diagnostic testing for the virus causing COVID-19 (67, 68). Although full validation on additional clinical samples is necessary before such an assay can be widely used, a few studies have evaluated this technique. These preliminary results demonstrate a promising approach to overcome the current bottlenecks that limit widespread testing.

Furthermore, the sequencing of the genome using salivary samples from COVID-19 patients could contribute in the incorporation of new targets (69), identification of the new SARS-CoV-2 variants of concern (as B.1.1.7 emerged in the United Kingdom, B.1.351 was first identified in South Africa, and P.1 emerged in Brazil) (70, 71), or even in the identification of critical mutations (72). In addition, a nanopore sequencing analysis of saliva suggests that host factors play a more important role in the clinical outcome than viral genetic variation (69), as demonstrated by emerging clinical studies (73).

## Final Remarks

These tests seem to be in agreement with FDA emergence approval, which includes a home collection of saliva to diagnose COVID-19 when indicated by a healthcare provider. Up until now, the FDA had authorized at least five salivary tests for COVID-19 diagnosis. The patients are also informed that a negative result is not a guarantee of the absence of COVID-19 infection. However, due to the high specificity of RT-PCR analysis, the detection of SARS-CoV-2 in saliva can be acceptable when the diagnostic test for COVID-19 is positive. We also highlight a substantial salivary detection of SARS-CoV-2 in patients with a negative nasopharyngeal swab (11.6%), indicating an expressive indication of false-negative results with gold standard specimens. Besides, the higher compliance to test for SARS-CoV-2 under reduced direct contact, requiring the collection of saliva, may contribute to an early diagnosis of COVID-19, resulting in optimal clinical care, encouraging isolation and reducing the spread of the disease. In this regard, the potential implementation of salivary SARS-CoV-2 diagnosis under a pandemic situation and social, health, economic, and educational issues due to COVID-19 is an additional challenge. These results support the potential of SARS-CoV-2 RNA as a biomarker for COVID-19, providing a self-collection, non-invasive, safe, and comfortable analysis, suitable to protect dentists, dental assistants, dental hygienists, and other frontline workers with self-collection and/or home collection saliva samples. Furthermore, we expect that salivary diagnostic devices for COVID-19 will continue to be used with austerity without excluding traditional gold standard specimens to detect SARS-CoV-2.

## Author Contributions

All authors listed have made a substantial, direct and intellectual contribution to the work, and approved it for publication.

## Conflict of Interest

RS-S, TC, LG, LC-S, and MM are inventors of patents related to the use of photonic devices for COVID-19 diagnosis using saliva and nasopharyngeal samples titled, Spectral profile for COVID-19 diagnostic, use of the same, method, system, and platform to COVID-19 diagnosis—BR 10 2020 010992 8. The remaining authors declare that the research was conducted in the absence of any commercial or financial relationships that could be construed as a potential conflict of interest.
